# Re-Birth After Coronary Bypass Graft Surgery: A Hermeneutic-Phenomenological Study

**DOI:** 10.5539/gjhs.v6n3p235

**Published:** 2014-03-31

**Authors:** Mohammad Abbasi, Nooredin Mohammadi, Alireza Nikbakht Nasrabadi, Suh Boudouin Fuh, Tahereh Sadeghi

**Affiliations:** 1 International Campus, Iran University of Medical Sciences, Tehran, Iran; 2 Center for Nursing Care Research, Department of Critical Care Nursing, School of Nursing and Midwifery, Iran University of Medical Sciences, Tehran, Iran; 3 School of Nursing and Midwifery, Tehran University of Medical Sciences, Tehran, Iran; 4 Department of Critical Care Nursing, School of Nursing and Midwifery, Tehran University of Medical Sciences, Tehran, Iran; 5 Tehran University of Medical Sciences, Tehran, Iran

**Keywords:** coronary artery bypass graft surgery, hermeneutic - phenomenological, lived experience, re-birth

## Abstract

Although coronary artery bypass graft surgery has significant effects on reducing the symptoms of coronary artery disease, there is not enough knowledge and understanding of lived experience of patients after surgery. Understanding lived experience of this group of patients would be helpful for healthcare staff to provide better services to the patients. The aim of this study was to describe with a deeper understanding, the lived experiences of patients after Coronary Artery Bypass Graft Surgery. Using a hermeneutic phenomenological approach and a Van-Manen analysis method, in-depth semi-structured interviews were conducted with eleven participants who had lived experienced of at least six months post - coronary artery bypass graft surgery. Re-birth was the main theme that emerged in the process of data analysis. This theme was derived from four sub-themes including “feels younger”, “vigorous heart”, “intrepid life” and “oriented to be healthy”. Life after a coronary artery bypass graft surgery is often appreciated as a re-birth by persons with these experiences as surgery did not only provide a feeling of wellness, but also added a sensation of youthfulness and improvement in the quality of life for these participants. In addition, they would actively participate in health promotional activities such as; adherence to medication and diet regimes, changes in lifestyle to maintain their health.

## 1. Introduction

Today, cardiovascular diseases (CVDs) are known as major health problems and the leading cause of high morbidity and mortality in both developed and developing countries (Roger et al.,2011). Despite advances in prevention, treatment and surgery in CVDs, the mortality rate of these diseases are still increasing significantly (Lopez, Ying, Poon, & Wai, 2007). In Iran like other developing countries, CVDs are the leading one of the major cause of death (Hadaegh et al.,2009). Based on published reports, about 45% of death in general population is due to CVDs (Dans et al., 2011) and coronary artery disease (CAD) is the most common CVDs in Iran (Azizi et al., 2009; Shakeri et al.,2012).

Although, there is a considerable progress in the non-surgical methods of management CAD, Coronary Artery Bypass Graft Surgery (CABGs) is still the most effective treatment to manage CAD. However, it needs to be pointed out that CABG is usually required when other medical and invasive treatments are not effective (Gersh, Sliwa, Mayosi, & Yusuf, 2010; Leegaard & Fagermoen, 2008). The number of CABGs is increasing around the world. In Iran, the number of CABGs which performed from 2002 to 2007 was reported between 21,000 and 30,000 cases (Babaee,2007).

CABGs has significant effects on reducing the symptoms of CAD; improving the health condition (Karlsson, Johansson, & Lidell, 2005); enhancing the daily life activities (Tolmie, Lindsay, & Belcher, 2006); improving the quality of life; increasing survival rate (Zhang et al.,2006); improving the life expectancy (Karlsson et al.,2005) and increasing the life-satisfaction in patients (Najmzadeh, 2007). Although CABGs has positive effects on patients, current literature reports that patients may still suffer from some consequences such as pain (Leegaard & Fagermoen, 2008); anxiety (Ivarsson, Larsson, & Sjöberg, 2004); fear (Karlsson et al.,2005); depression, anger (Leegaard & Fagermoen, 2008) and hospital re-admissions (Mousavi, Sabzevari, Abbaszade, & Hosseinnakhaie, 2011) in post-surgical period.

Although there are plenty studies which have focused on the physical and psychological complications of CABGs and their consequences on patients’ life, there is limited knowledge about the experiences of patients after CABGs. Therefore, the lived experience of patients after coronary bypass is not well known. Understanding the lived experience of this group of patients helps health care professionals to provide a better service to these patients. This study was aimed to explore a deeper understanding of the lived experience of patients after CABGs.

## 2. Methods

An interpretive phenomenology inquiry with the Heidegger’s approach was used in this study. Van-Manen’s six methodological activities were used to guide the researcher to conduct the study (Van Manen, 1990). Table 1 summarizes the six activities of van Manen’s which used in the study.

**Table 1:** van Manen’s method of doing phenomenological study and its use in the study

van Manen’s methodical activities	Researchers’ activities
Turning to the nature of lived experience	selected participants having the life experience with CABG to acquire humanistic experiences
Investigating experience as we live it	collect data through people having the lived experience with CABG
Reflecting on essential themes	by using thematic analysis
Hermeneutic phenomenological writing	writing and rewriting to create a phenomenological text
Maintaining a strong and oriented nursing relation to the phenomenon	Discussing the themes in relation to phenomena
Balancing the research context by considering parts and whole	movement between transcripts and themes in relation to CABG lived experience

### 2.1 Participants

Participants were made up of seven men and four women with an average age of 59.60 ± 3.77 years, married and living with their families. Inclusion criteria for participation were patient who had lived experiences with CABGs of at least six months and had no mental problems and malignant disease at the time of their participation in the study.

The first contact of the researcher with the patients was in the follow-up clinic. When the potential participants attended in the clinic, the researcher assessed their eligibility to participate in the study. Then the research purpose of the study was explained to the eligible participants and they were encouraged to participate in the study. The recruited participants provided their contact detail to the researcher to schedule appointments for the introductory meeting and interviews at a place and time which they were convenient to both.

Therefore the researcher had an introductory session with the participants before the interview. The purpose of the introductory meeting was to establish trust between the participants and the researcher by answering all queries with regards to the study. At the end of introductory meeting, the researcher left an informed consent form to the participants and organized the interview session with the participants.

### 2.2 Data Collection

In-depth semi-structured interviews were conducted by the principle researcher to collect data. Before the interview, the researcher collected the signed consent form from all participants. The researcher again explained the objectives of the study to the participants and answered to their questions and concerns. Individual face-to-face interviews were performed with participants in a quiet room at the treatment centers or at the participants’ home. All interviews were performed in native language (Persian). The interviews lasted on average between 55 to 70 minutes. During the interviews, participants were encouraged to express their lived experiences after the CABGs as a story. In situations where the researcher did not accurately understand the participant, he probed more into the story and asked questions such as; “… can you give me an example?” or “What did you mean by…?” for a better understanding. All interviews were conducted by the principle researcher and they were recorded on a tape recorder and some notes were also taken in writing

### 2.3 Data Analysis

Analysis of the data was done on texts obtained from the interviews. First, the researchers listened to the audio interviews for several times, and wrote them word verbatim on paper. To ensured texts accuracy, they listened and read text files simultaneously. The researchers then extracted themes or units of meaning using a holistic approach, selective approach and detailed approach. They read the text over and over until a comprehensive understanding of the participants’ experience was attained. Words, phrases and sentences from the participants’ narrations were separated to extract the major themes of their lived experience with CABGs.

The validity of this study was achieved through an effective trust-based relationship that was established between participants and the researchers. Data from interviews were presented to the participants after analysis and their corrective feedbacks were considered. The extracted themes were discussed with most of the participants for their approval. Furthermore, every step of the study process had been checked with supervisors and experts to get their feedbacks to make sure the process of the study is going in right direction.

### 2.4 Ethical Consideration

This study was approved by the Ethics Committee of Tehran University of Medical Sciences.

## 3. Findings

Open heart surgery was a landmark for the participants to categorize their life into two separate phases, the life before CABGs and the life after CABGs. During interviews, the participants were expressing their lived experiences while they tried to compare their experiences in these two phases. For example, one of the participants stated: *“my life after surgery is extremely different with before surgery”*. Another participant expressed, *‘To be honest, I am another person after surgery’*.

The reason why the participants were identifying these two separate phases in their life was the changes in their physical activities and psychological status. The significance of those changes caused that the participants believe that they were born again. Therefore the theme “re-birth” was extracted as the main theme in this study. As one of participants said that; “*Heart surgery was an opportunity given to me to live again, And to start a new life with the new heart. I was reborn and it was as a gift from surgery”*.

The theme “re-birth” comes from the participants’ perception of changes in their heart after surgery. For some of the participants, heart surgery was a revolution of their life and they supposed their heart is like a child’s heart. One of the participants pointed out, “*Someone who undergoes an open heart surgery will have a heart like child’s heart”*. The theme “re-birth” was derived from four following sub-themes. These sub-themes are “feel younger”, “vigorous heart”, “intrepid life” and “oriented to be healthy”.

### 3.1 Theme 1. Feel Younger

For the participants of the study, the limitation in daily life activity due to CAD was a reason which led to an undesirable feeling in the phase of before CABGs. Some of the participants said that they were feeling older and weaker in this phase. One of the participants stated:

“*Before undergoing for surgery, I was thinking that I am going to be old earlier because my ability to do works was going to be less and less, like a very old woman while I was just in the middle of 50’s*.”

Participants added the life after surgery was accompanied with a positive feeling of being younger. This feeling was a consequence of physical and psychological recovery and improving daily life activity among the participants. One of the participants stated “*After the heart surgery, I felt physically and mentally recovered. I had sensations of freshness, youthfulness and was highly spirited. I felt younger than I was”*.

### 3.2 Theme 2. Vigorous Heart

The participants of study pointed out that they had a fatigued heart in the phase of before CABGs. One of the participants told “*My heart was sick like an out of order car. It needed to be fix to work appropriately”*. Participants believed they had inactive life style before CABGs because of their heart was fatigued and had no sufficient ability to perform properly its function. For example, one of the participants said *“My heart was not working well before surgery. That’s why I was staying home most times when my family was going somewhere”*.

Open heart Surgery for the participants was not only a treatment to manage their health problem, but also it was an intervention which reconstructed their exhausted heart and improved its function. Participants thought they have a healthy heart in the post CABGs life. The following statement is a typical statement which has been taken from one of the participants. *“The surgery repaired my sick heart and now I’m living with a healthy heart”*.

### 3.3 Theme 3. Intrepid Life

Life before surgery was a life with full of fear and apprehension for most of the participants. They said that they were often afraid for a heart attack and sudden death in the phase of before the surgery. A participant was talking about his fears and concerns narrated: “*Before, I was always afraid my heart is stop suddenly and I was often worried about a heart attack or death”*. Some other participants expressed the fear of heart attack and sudden death was even when they were asleep and consequently they suffered from insomnia and fatigue. The following quotation is from one of the participants. *“Before surgery, I was so worried and anxious and very sad too. Sometimes at night, I couldn’t sleep because I was scared to die when I am asleep”*. Fear of heart attack also forced the participants to avoid social activities. Another participant stated:

*“Before the heart surgery when I was going out, I was always scared of falling suddenly and nobody would recognize me* [due to heart attack]. *So I always carried my identification card with myself to help people identify me if I unconciseness”*.

Although participants narrated their fear and concern during interviews, they insisted that there was no fear of heart attack and sudden death after the surgery. For example, this excerpt is from one of the participants. *“.. I am not scared to go out alone any more*”. Although participants were experiencing fear in the phase of post CABGs, the nature of fear was totally different with the phase of before CABGs. For instance, the same participant added *“When I was discharged from the hospital, I was always scared to cough hard because of stitches’*. Another participant added *‘I was scared for a long time that my grafts not working well”*.

### 3.4 Theme 4. Oriented to Be Healthy

Participants expressed that they had little attention to their medical orders and diet regimens before surgery, although some of them had good knowledge in this regards. As an example, one participant said, *“I was not sensitivity to my diet before surgery. …I used to have salty food. However I was informed by my doctor that it was not good for me”*. Therefore participants’ adherence to treatment plan was weak in the life before CABGs.

For participants in this study, the life after CABGs was accompanied with more adherences to the medical treatment plans. One of the participants emphasized the importance of following medical instructions and believed that following the instructions can help to prevent the re-infection of CAD. The following statement is from one of the participants in this study. “*I’m caring myself now. I follow my instructions in order to not have the heart problems again”*. Thus the participants were eagerly following their medication and dietary regimens after surgery in order to maintain and promote their health condition.

## 4. Discussion

Re-birth, as the main theme of this study, was obtained from the participants experiences. Re-birth does not mean that all of the participants believed that their organs have been born a second time, but this theme was chosen due to the importance and role of the heart in human life according to the participants. After CABGs, participants felt that they had regained their health and this is because before CABGs they felt dizziness, weakness, chest pain and dyspnea. They had problems in performing their daily activities, but many of these problems decreased after the CABGs, relieving participants of their discomforts and enabling them to resume their activities normally. CABGs does not only provide a feeling of wellness, but also adds a sensation of youthfulness and improves the quality of life as demonstrated in many studies (Najmzadeh, 2007; Tolmie, 2006; Zhang,2006).

Perhaps one of the factors that have been effective in the feeling of youthfulness in participants was the improved quality of life after CABGs. Participants were without difficulty after CABGs in terms of mobility and daily activities thus considering their quality of life improved. Hawkes and colleagues reported that patients resume their activities after CABGs (Hawkes, Nowak, Bidstrup, & Speare, 2006). The increased activity and personal tasks make sense of freshness and high spirits in participants. Lindquist and colleagues reported that CABGs improves the quality of life in domains of social, physical, emotional functioning and satisfaction in their lives (Lindquist et al.,2003). Participants believed that their hearts were reconstructed after the CABGs. The reason for this believes was that since the participants returned to their normal daily activities after the CABGs and did not have chest pain and dyspnea anymore, they knew that they are living with a healthy heart. They associated chest pain and dyspnea with heart disease, so the relief of chest pain and dyspnea was a gift of CABGs. To them, after the CABGs, they were certain that they will not have heart problems anymore as their hearts have been rescued of pain and now they live with a healthy and trustworthy heart. This agrees with the findings of study of Tolmie, Lindsay and Belcher (2006), that CABGs helps the patients to be free of angina symptoms and improves on their quality of life.

The recovery period after the CABGs is a dynamic process and participants have a unique experience during this period. They may express fear immediately after the CABGs. Fears and concerns after CABGs have been noted in many studies (Mousavi et al., 2011; Schou, 2008; Bergvik, 2008). In this study, participants had experienced fears and concerns too, but the nature of these fears and concerns were different. They were not afraid about heart attack and sudden death but were rather afraid complications they think may arise from dehiscence of the coronary sutures due to them sneezing, having a strong cough or frequent mobility. Here one can realize the importance of education in the period after surgery. Thus, if the patients do not receive adequate and appropriate discharge training after the CABGs, they may not perform the post-operative exercises such as deep breathing and effective coughing; and consequently suffer from some complications such as atelectasis (Hulzebos et al., 2006; Jensen, 2007).

## 5. Conclusion

CABGs provided the opportunity for the acquisition and promotion of good health, free of cardiovascular problems for the participants of this study. They acknowledged that they had no proper lifestyle immediately they knew they had a heart disease. However, they have regained their lost health after the CABGs. Thus they have tried to be involved actively in effective measures to maintain their health. They concluded by stating that a modification of their lifestyle with programs such as regular exercises and a modified diet of low salt and low fat consumption would help them prevent another episode of heart disease. More so, they requested nutritional consultation support for a healthy diet, as they try to lose weight too. Stress management activities such as avoiding stressful environments and coping with others were also pinpointed as necessary for their health. These recommendations agree with Lindsay and Colleagues findings that some participants identified some measures to protect their health (Lindsay, Hanlon, Smith, & Belcher, 2003). Goldsmith and Colleagues also reported lifestyle changes after a cardiac event (Goldsmith, Lindholm, & Bute, 2006).
